# 2-Amino-3-[(*E*)-(2-hy­droxy-3-methyl­benzyl­idene)amino]­but-2-ene­dinitrile

**DOI:** 10.1107/S1600536812015243

**Published:** 2012-04-18

**Authors:** Elham S. Aazam, Orhan Büyükgüngör

**Affiliations:** a Department of Chemistry, Girls Section, King Abdulaziz University, PO Box 6171, Jeddah 21442, Saudi Arabia; b Ondokuz Mayıs University, Arts and Sciences Faculty, Department of Physics, 55139-Samsun, Turkey

## Abstract

The title compound, C_12_H_10_N_4_O, is a Schiff base obtained from the condensation of diamino­maleonitrile and 2-hy­droxy-3-methyl­benzaldehyde. The mol­ecule is roughly planar, with an r.m.s. deviation of 0.0354 Å, and adopts the phenol–imine tautomeric form. An intra­molecular O—H⋯N hydrogen bond involving the O—H group and the azomethine N atom generates an *S*(6) ring. In the crystal, there are two N—H⋯N hydrogen bonds.

## Related literature

For the biological properties of Schiff bases see: Da Silva *et al.* (2011 ▶) and for their use in coordination chemistry, see: Aazam *et al.* (2011 ▶); Kargar *et al.* (2009 ▶); Yeap *et al.* (2009 ▶). For graph-set notation, see: Bernstein *et al.*, (1995 ▶). For related structures, see: Aazam & Büyükgüngör (2010 ▶); Hökelek *et al.* (2000 ▶); Odabaşoğlu *et al.* (2005 ▶); Rivera *et al.* (2006 ▶). 
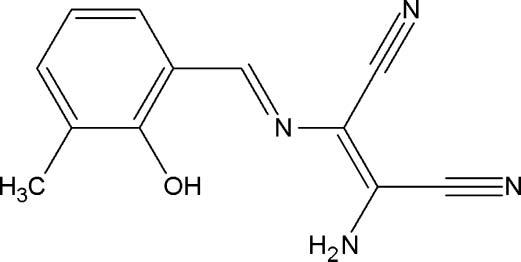


## Experimental

### 

#### Crystal data

C_12_H_10_N_4_O
*M* *_r_* = 226.24
Monoclinic, 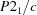

*a* = 6.9041 (6) Å
*b* = 11.8791 (7) Å
*c* = 14.0282 (11) Å
β = 101.600 (7)°
*V* = 1127.02 (15) Å^3^
*Z* = 4
Mo *K*α radiation
μ = 0.09 mm^−1^
*T* = 296 K
0.76 × 0.48 × 0.03 mm

#### Data collection

Stoe IPDS 2 diffractometer
Absorption correction: integration (*X-RED32*; Stoe & Cie, 2002 ▶) *T*_min_ = 0.949, *T*_max_ = 0.996
16504 measured reflections
2336 independent reflections
1700 reflections with *I* > 2σ(*I*)
*R*_int_ = 0.054

#### Refinement

*R*[*F* ^2^ > 2σ(*F* ^2^)] = 0.059
*wR*(*F* ^2^) = 0.132
*S* = 1.14
2336 reflections
168 parameters
H atoms treated by a mixture of independent and constrained refinement
Δρ_max_ = 0.15 e Å^−3^
Δρ_min_ = −0.16 e Å^−3^

### 

Data collection: *X-AREA* (Stoe & Cie, 2002 ▶); cell refinement: *X-AREA*; data reduction: *X-RED32* (Stoe & Cie, 2001 ▶); program(s) used to solve structure: *SHELXS97* (Sheldrick, 2008 ▶); program(s) used to refine structure: *SHELXL97* (Sheldrick, 2008 ▶); molecular graphics: *ORTEP-3 for Windows* (Farrugia, 1997 ▶); software used to prepare material for publication: *WinGX* (Farrugia, 1999 ▶).

## Supplementary Material

Crystal structure: contains datablock(s) I, global. DOI: 10.1107/S1600536812015243/go2051sup1.cif

Structure factors: contains datablock(s) I. DOI: 10.1107/S1600536812015243/go2051Isup2.hkl

Supplementary material file. DOI: 10.1107/S1600536812015243/go2051Isup3.cml

Additional supplementary materials:  crystallographic information; 3D view; checkCIF report

## Figures and Tables

**Table 1:** Hydrogen-bond geometry (Å, °)

*D*—H⋯*A*	*D*—H	H⋯*A*	*D*⋯*A*	*D*—H⋯*A*
O1—H1⋯N1	0.88 (3)	1.83 (3)	2.643 (2)	153 (3)
N2—H2*A*⋯N4^i^	0.89 (3)	2.40 (3)	3.156 (3)	142 (2)
N2—H2*B*⋯N3^ii^	0.88 (3)	2.26 (3)	3.098 (3)	159 (2)

Symmetry codes: (i) 
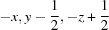
; (ii) 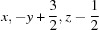.

**Fig. 1.**
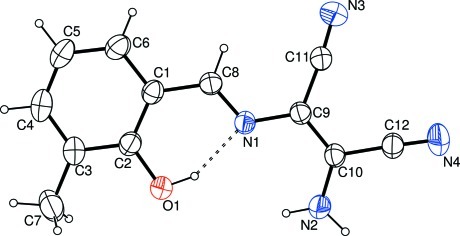
The molecular structure of the title compound, showing 50% probability displacement ellipsoids and atomic numbering.

**Fig. 2.**
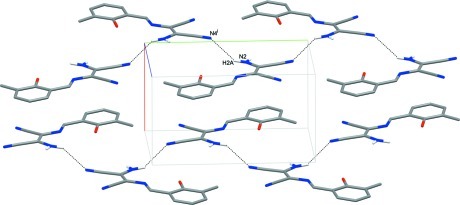
Part of the crystal structure of the title compound, showing the formation of C(5) chains parallel to the b axis (i; -x, y - 1/2, -z + 1/2). Hydrogen bonds are indicated by dashed lines.

**Fig. 3.**
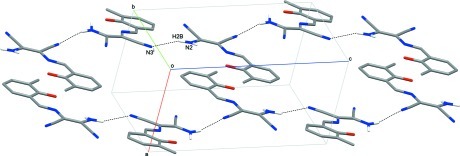
Part of the crystal structure of the title compound, showing the formation of C(6) chains parallel to the c axis (i; x, -y + 3/2, z - 1/2). Hydrogen bonds are indicated by dashed lines.

**Fig. 4.**
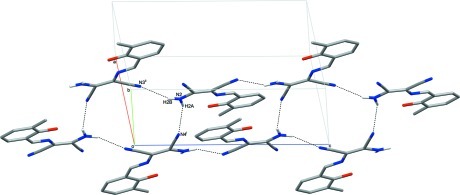
Part of the crystal structure of the title compound, showing the formation of R44(18) and R44(22) rings. Hydrogen bonds are indicated by dashed lines. (Symmetry codes as in Table 1)

## supplementary crystallographic information

### Comment

Tetrameric HCN (diaminomaleonitrile, DAMN) is one of the most versatile reagents in organic chemistry. It has been used as a precursor for producing nucleotides and for synthesizing a wide variety of heterocyclic compounds. These compounds are important as synthetic intermediates and they are also used in pharmacology (Da Silva *et al.*, 2011; Rivera *et al.*, 2006).

Schiff bases derived from DAMN have also been used as versatile ligands in coordination chemistry (Aazam *et al.*, 2011; Kargar *et al.*, 2009; Yeap *et al.*, 2009). There are two types of intra-molecular hydrogen bonds in Schiff bases, which may be stabilized either in keto-amine (N—H···O hydrogen bond) (Hökelek *et al.*, 2000) or phenol-imine (N···H—O hydrogen bond) tautomeric forms (Odabaşoǧlu *et al.*, 2005; Aazam & Büyükgüngör, 2010). The present X-ray investigation shows that the title compound is a Schiff base and exists in the phenol-imine form in the solid-state.

The molecular structure of the title compound is shown in Figure 1. An intramolecular O1—H1···N1 hydrogen bond, a characteristic hydrogen bond for Schiff bases, leads to the formation of a S(6) six-membered ring (Figure 1) (Bernstein *et al.*, 1995).

The N2—H2A···N4^i^ (i; -*x*, *y* - 1/2, -*z* + 1/2) and N2—H2B···N3^ii^ (ii; *x*, -*y* + 3/2, *z* - 1/2) hydrogen bonds generate a C(5) zigzag chain running parallel to the *b* axis and a C(6) chain running parallel to the *c* axis, respectively, (Figure 2–3). The intersection of the C(5) and C(6) chains produce alternating *R*_4_^4^(18) and *R*_4_^4^(22) ring motives, (Figure 4), (Bernstein *et al.*, 1995) which link the molecules into corrugated sheets which lie in the *b,c* plane. Details of the hydrogen bonds are given in Table 1.

### Refinement

The H atoms bonded to oxygen and nitrogen atoms were located in Fourier map and refined isotropically. Other hydrogen atoms were positioned geometrically and treated using a riding model, fixing bond lengths at 0.93 and 0.96 Å for CH (aromatic) and CH_3_, respectively. The displacement parameters of the H atoms were constrained with *U*_iso_(H) = 1.2*U*_eq_ (aromatic and methyl C).

### Crystal data

**Table:**

C_12_H_10_N_4_O	*F*(000) = 472
*M**_r_* = 226.24	*D*_x_ = 1.333 Mg m^−^^3^
Monoclinic, *P*2_1_/*c*	Mo *K*α radiation, λ = 0.71073 Å
Hall symbol: -P 2ybc	Cell parameters from 16504 reflections
*a* = 6.9041 (6) Å	θ = 2.3–28.0°
*b* = 11.8791 (7) Å	µ = 0.09 mm^−^^1^
*c* = 14.0282 (11) Å	*T* = 296 K
β = 101.600 (7)°	Plate, brown
*V* = 1127.02 (15) Å^3^	0.76 × 0.48 × 0.03 mm
*Z* = 4	

### Data collection

**Table:**

Stoe IPDS 2 diffractometer	2336 independent reflections
Radiation source: fine-focus sealed tube	1700 reflections with *I* > 2σ(*I*)
Graphite monochromator	*R*_int_ = 0.054
rotation method scans	θ_max_ = 26.5°, θ_min_ = 2.3°
Absorption correction: integration (*X-RED32*; Stoe & Cie, 2002)	*h* = −8→8
*T*_min_ = 0.949, *T*_max_ = 0.996	*k* = −14→14
16504 measured reflections	*l* = −17→17

### Refinement

**Table:**

Refinement on *F*^2^	Secondary atom site location: difference Fourier map
Least-squares matrix: full	Hydrogen site location: inferred from neighbouring sites
*R*[*F*^2^ > 2σ(*F*^2^)] = 0.059	H atoms treated by a mixture of independent and constrained refinement
*wR*(*F*^2^) = 0.132	*w* = 1/[σ^2^(*F*_o_^2^) + (0.0644*P*)^2^ + 0.0085*P*] where *P* = (*F*_o_^2^ + 2*F*_c_^2^)/3
*S* = 1.14	(Δ/σ)_max_ < 0.001
2336 reflections	Δρ_max_ = 0.15 e Å^−^^3^
168 parameters	Δρ_min_ = −0.16 e Å^−^^3^
0 restraints	Extinction correction: *SHELXL97* (Sheldrick, 2008), Fc^*^=kFc[1+0.001xFc^2^λ^3^/sin(2θ)]^-1/4^
Primary atom site location: structure-invariant direct methods	Extinction coefficient: 0.008 (2)

### Special details

**Table:**

Geometry. All e.s.d.'s (except the e.s.d. in the dihedral angle between two l.s. planes) are estimated using the full covariance matrix. The cell e.s.d.'s are taken into account individually in the estimation of e.s.d.'s in distances, angles and torsion angles; correlations between e.s.d.'s in cell parameters are only used when they are defined by crystal symmetry. An approximate (isotropic) treatment of cell e.s.d.'s is used for estimating e.s.d.'s involving l.s. planes.
Refinement. Refinement of *F*^2^ against ALL reflections. The weighted *R*-factor *wR* and goodness of fit *S* are based on *F*^2^, conventional *R*-factors *R* are based on *F*, with *F* set to zero for negative *F*^2^. The threshold expression of *F*^2^ > σ(*F*^2^) is used only for calculating *R*-factors(gt) *etc*. and is not relevant to the choice of reflections for refinement. *R*-factors based on *F*^2^ are statistically about twice as large as those based on *F*, and *R*- factors based on ALL data will be even larger.

### Fractional atomic coordinates and isotropic or equivalent isotropic displacement parameters (Å^2^)

**Table:**

	*x*	*y*	*z*	*U*_iso_*/*U*_eq_	
C1	0.2927 (3)	0.39788 (16)	0.57527 (14)	0.0412 (5)	
C2	0.2741 (3)	0.30861 (18)	0.50843 (15)	0.0451 (5)	
C3	0.2980 (3)	0.19643 (18)	0.53978 (17)	0.0507 (5)	
C4	0.3421 (3)	0.1774 (2)	0.63866 (19)	0.0579 (6)	
H4	0.3581	0.1036	0.6609	0.069*	
C5	0.3637 (3)	0.2639 (2)	0.70634 (17)	0.0597 (6)	
H5	0.3951	0.2480	0.7726	0.072*	
C6	0.3382 (3)	0.3731 (2)	0.67447 (16)	0.0518 (5)	
H6	0.3514	0.4313	0.7196	0.062*	
C7	0.2734 (4)	0.1024 (2)	0.4671 (2)	0.0730 (7)	
H7A	0.3735	0.1081	0.4287	0.088*	
H7B	0.1452	0.1073	0.4254	0.088*	
H7C	0.2860	0.0315	0.5006	0.088*	
C8	0.2653 (3)	0.51404 (16)	0.54458 (14)	0.0426 (5)	
H8	0.2785	0.5698	0.5920	0.051*	
C9	0.2013 (3)	0.65633 (15)	0.42752 (14)	0.0393 (5)	
C10	0.1644 (3)	0.68409 (16)	0.33180 (14)	0.0405 (5)	
C11	0.2178 (3)	0.74210 (17)	0.50067 (15)	0.0461 (5)	
C12	0.1464 (3)	0.80158 (18)	0.30383 (15)	0.0482 (5)	
N1	0.2236 (2)	0.54359 (13)	0.45456 (11)	0.0405 (4)	
N2	0.1385 (3)	0.61018 (17)	0.25795 (15)	0.0563 (5)	
H2A	0.112 (4)	0.538 (3)	0.2695 (19)	0.077 (8)*	
H2B	0.130 (4)	0.637 (2)	0.199 (2)	0.076 (8)*	
N3	0.2305 (3)	0.80465 (17)	0.56309 (15)	0.0664 (6)	
N4	0.1280 (3)	0.89245 (17)	0.27921 (17)	0.0709 (6)	
O1	0.2304 (3)	0.32719 (15)	0.41171 (12)	0.0668 (5)	
H1	0.217 (4)	0.400 (3)	0.405 (2)	0.081 (9)*	

### Atomic displacement parameters (Å^2^)

**Table:**

	*U*^11^	*U*^22^	*U*^33^	*U*^12^	*U*^13^	*U*^23^
C1	0.0386 (10)	0.0447 (11)	0.0419 (11)	0.0035 (8)	0.0116 (8)	0.0071 (9)
C2	0.0471 (11)	0.0469 (12)	0.0413 (12)	0.0045 (9)	0.0090 (9)	0.0061 (9)
C3	0.0485 (12)	0.0429 (12)	0.0594 (14)	0.0028 (9)	0.0076 (10)	0.0095 (10)
C4	0.0520 (13)	0.0504 (13)	0.0705 (17)	0.0023 (10)	0.0108 (11)	0.0236 (12)
C5	0.0649 (14)	0.0679 (16)	0.0466 (13)	0.0040 (11)	0.0120 (11)	0.0215 (12)
C6	0.0563 (13)	0.0597 (14)	0.0417 (12)	0.0015 (10)	0.0149 (10)	0.0064 (10)
C7	0.0825 (17)	0.0444 (13)	0.0863 (19)	0.0090 (12)	0.0027 (14)	−0.0023 (13)
C8	0.0473 (11)	0.0408 (11)	0.0415 (12)	0.0017 (8)	0.0135 (8)	0.0006 (9)
C9	0.0416 (10)	0.0355 (10)	0.0421 (11)	−0.0004 (8)	0.0113 (8)	0.0004 (8)
C10	0.0430 (10)	0.0345 (10)	0.0436 (12)	−0.0030 (8)	0.0077 (8)	0.0026 (8)
C11	0.0566 (13)	0.0382 (11)	0.0446 (12)	0.0035 (9)	0.0127 (10)	0.0029 (10)
C12	0.0548 (12)	0.0404 (12)	0.0482 (12)	−0.0029 (9)	0.0072 (9)	0.0055 (10)
N1	0.0450 (9)	0.0367 (8)	0.0411 (10)	0.0015 (7)	0.0114 (7)	0.0039 (7)
N2	0.0836 (14)	0.0420 (11)	0.0416 (11)	−0.0110 (10)	0.0084 (9)	0.0022 (9)
N3	0.0917 (15)	0.0538 (12)	0.0540 (12)	0.0047 (10)	0.0151 (11)	−0.0096 (10)
N4	0.0901 (15)	0.0433 (11)	0.0779 (15)	0.0048 (10)	0.0134 (12)	0.0155 (10)
O1	0.1120 (14)	0.0444 (9)	0.0415 (10)	0.0164 (9)	0.0098 (9)	0.0023 (7)

### Geometric parameters (Å, º)

**Table:**

C1—C6	1.395 (3)	C7—H7C	0.9600
C1—C2	1.404 (3)	C8—N1	1.286 (2)
C1—C8	1.447 (3)	C8—H8	0.9300
C2—O1	1.348 (3)	C9—C10	1.356 (3)
C2—C3	1.403 (3)	C9—N1	1.392 (2)
C3—C4	1.378 (3)	C9—C11	1.434 (3)
C3—C7	1.499 (3)	C10—N2	1.342 (3)
C4—C5	1.386 (4)	C10—C12	1.448 (3)
C4—H4	0.9300	C11—N3	1.138 (3)
C5—C6	1.372 (3)	C12—N4	1.133 (3)
C5—H5	0.9300	N2—H2A	0.89 (3)
C6—H6	0.9300	N2—H2B	0.88 (3)
C7—H7A	0.9600	O1—H1	0.88 (3)
C7—H7B	0.9600		
			
C6—C1—C2	118.59 (18)	H7A—C7—H7B	109.5
C6—C1—C8	119.22 (19)	C3—C7—H7C	109.5
C2—C1—C8	122.19 (18)	H7A—C7—H7C	109.5
O1—C2—C3	117.37 (19)	H7B—C7—H7C	109.5
O1—C2—C1	121.37 (18)	N1—C8—C1	122.86 (18)
C3—C2—C1	121.26 (19)	N1—C8—H8	118.6
C4—C3—C2	117.4 (2)	C1—C8—H8	118.6
C4—C3—C7	122.3 (2)	C10—C9—N1	119.49 (17)
C2—C3—C7	120.3 (2)	C10—C9—C11	120.52 (17)
C3—C4—C5	122.7 (2)	N1—C9—C11	119.99 (17)
C3—C4—H4	118.7	N2—C10—C9	125.07 (19)
C5—C4—H4	118.7	N2—C10—C12	115.50 (19)
C6—C5—C4	119.2 (2)	C9—C10—C12	119.43 (18)
C6—C5—H5	120.4	N3—C11—C9	175.5 (2)
C4—C5—H5	120.4	N4—C12—C10	177.7 (2)
C5—C6—C1	120.9 (2)	C8—N1—C9	121.38 (16)
C5—C6—H6	119.6	C10—N2—H2A	118.9 (17)
C1—C6—H6	119.6	C10—N2—H2B	117.7 (18)
C3—C7—H7A	109.5	H2A—N2—H2B	123 (2)
C3—C7—H7B	109.5	C2—O1—H1	105.2 (19)
			
C6—C1—C2—O1	179.99 (18)	C2—C1—C6—C5	−0.2 (3)
C8—C1—C2—O1	0.3 (3)	C8—C1—C6—C5	179.5 (2)
C6—C1—C2—C3	0.8 (3)	C6—C1—C8—N1	179.86 (18)
C8—C1—C2—C3	−178.93 (19)	C2—C1—C8—N1	−0.4 (3)
O1—C2—C3—C4	−179.80 (19)	N1—C9—C10—N2	2.9 (3)
C1—C2—C3—C4	−0.6 (3)	C11—C9—C10—N2	−177.18 (19)
O1—C2—C3—C7	−0.5 (3)	N1—C9—C10—C12	−178.38 (17)
C1—C2—C3—C7	178.8 (2)	C11—C9—C10—C12	1.5 (3)
C2—C3—C4—C5	−0.2 (3)	C1—C8—N1—C9	−178.97 (18)
C7—C3—C4—C5	−179.5 (2)	C10—C9—N1—C8	177.62 (18)
C3—C4—C5—C6	0.8 (3)	C11—C9—N1—C8	−2.3 (3)
C4—C5—C6—C1	−0.5 (3)		

### Hydrogen-bond geometry (Å, º)

**Table:**

*D*—H···*A*	*D*—H	H···*A*	*D*···*A*	*D*—H···*A*
O1—H1···N1	0.88 (3)	1.83 (3)	2.643 (2)	153 (3)
N2—H2*A*···N4^i^	0.89 (3)	2.40 (3)	3.156 (3)	142 (2)
N2—H2*B*···N3^ii^	0.88 (3)	2.26 (3)	3.098 (3)	159 (2)

Symmetry codes: (i) −*x*, *y*−1/2, −*z*+1/2; (ii) *x*, −*y*+3/2, *z*−1/2.
